# Human and Canine Pulmonary Blastomycosis, North Carolina, 2001–2002

**DOI:** 10.3201/eid1208.050781

**Published:** 2006-08

**Authors:** Pia D.M. MacDonald, Rick L. Langley, Susan R. Gerkin, Michelle R. Torok, J. Newton MacCormack

**Affiliations:** * Centers for Disease Control and Prevention, Atlanta, Georgia, USA;; † North Carolina Division of Public Health, Raleigh, North Carolina, USA;; ‡ East Carolina University, Greenville, North Carolina, USA;; § University of North Carolina, Chapel Hill, North Carolina, USA

**Keywords:** Blastomycosis, Blastomyces dermatitidis, humans, canine diseases, disease outbreaks, lung disease, fungal, North Carolina, dispatch

## Abstract

We investigated a cluster of blastomycosis in 8 humans and 4 dogs in a rural North Carolina community. Delayed diagnosis, difficulty isolating *Blastomyces dermatitidis* in nature, and lack of a sensitive and specific test to assess exposure make outbreaks of this disease difficult to study.

*Blastomyces dermatitidis* is the etiologic agent of blastomycosis, a fungal disease that affects humans and animals, particularly dogs. The ecologic niche for *B. dermatitidis* is not fully understood, although research suggests acquisition of blastomycosis may be associated with environmental conditions such as sandy soil, organic matter, waterways, and earth-disturbing activities (1–4). In North America, the southeastern and south central states and parts of the Midwest, Canada, and New York have been identified as areas where the disease is endemic (1). Most sporadic and outbreak cases have also been reported from these regions (4). In states where blastomycosis is reportable, the annual incidence is 1.3–1.4 cases per 100,000 persons. However, areas of hyperendemicity can have rates of up to 41.9 cases per 100,000 persons (5). Furthermore, incidence of this disease has been increasing in certain regions (6). This report describes a recent cluster of human and canine pulmonary blastomycosis that occurred in rural North Carolina.

## The Study

From November 2001 to February 2002, pulmonary blastomycosis was diagnosed in 8 residents of a small town in Duplin County, located in eastern North Carolina. In contrast, 1 human case of pulmonary blastomycosis was identified in Duplin County from January 1995 through June 2001. Four patients attended the same school. Three cases of canine blastomycosis with onset in late December 2001 and January 2002 were also diagnosed by a veterinarian in the same town; canine blastomycosis had not been diagnosed for at least 7 years before 2002.

Active case finding was conducted from September 2001 to February 2002. Duplin County death certificate and hospital discharge information was reviewed for blastomycosis. Clinical laboratories and infection control programs for major hospitals in eastern North Carolina were queried for blastomycosis or unusual pneumonia cases. Workers at high occupational risk for blastomycosis (e.g., construction crews, cemetery workers, and county road scrapers) were also contacted. All county veterinarians were contacted to identify additional canine cases.

To explore commonalities among cases, surviving patients or family members of dead patients were interviewed by using a standardized questionnaire. Information was obtained on patient demographics, recreational and work-related activities, medical history, and pet ownership. Information was limited to the hospital record for 1 patient. Because half of the patients attended the same school, an environmental assessment of the school grounds was performed. Finally, historical climatic data were obtained to compare conditions during and before the outbreak. Demographic and clinical data for human cases are summarized in Table 1.

**Table 1:** Demographic and clinical features of human blastomycosis patients, Duplin County, North Carolina, 2001

Feature	Value
Median age, y (range)	25 (15–82)
Race, no. (%)	
African American	5 (63)
White	3 (37)
Sex, no. (%)	
Male	6 (75)
Clinical signs and symptoms, no. (%)	
Cough	5 (71)
Fever	6 (86)
Chest pain	6 (86)
Shortness of breath	4 (57)
Clinical outcomes, no. (%)	
Hospitalized	8 (100)
Pneumonia diagnosed	8 (100)
Failed antimicrobial drug treatment	8 (100)
Treated with itraconazole*	6 (75)
Survived	7 (87)

*One patient with multiple underlying medical problems was treated with amphotericin B; another patient was treated with fluconazole.

The epidemic plotting of this outbreak suggests ongoing exposure (Figure), as was the experience in previous North Carolina blastomycosis outbreaks (7,8). However, neither the epidemiologic investigation nor the environmental assessment showed a common source for human and canine exposure. The only commonality among the students was involvement with different outdoor after-school activities. The remaining patients did not frequent the school grounds. Two of the adult patients lived within 0.4 km of each other. Otherwise, no commonalities were noted in hobbies, occupation, or recreational activities between patients. None of the canine cases had contact with the patients or other infected dogs. One dog was primarily an indoor dog; another was kept in an outside run for 3 months before illness caused by suspected rabies exposure. Symptom onset was first recognized in humans, although the epidemic curve suggests that human and canine patients were exposed during the same period.

**Figure:**
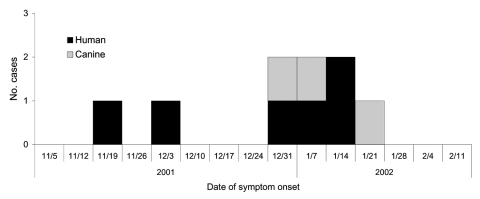
Epidemic curve of a human and canine pulmonary blastomycosis outbreak, North Carolina, 2001–2002.

Several environmental conditions, such as soil type and pH, proximity to waterways, elevation of nearest waterway, temperature, precipitation, and earth-disturbing activities might facilitate growth of *B. dermatitidis* and have been associated with blastomycosis (1–4,9). Duplin County is located in the middle coastal plain region of the state, 34 m above sea level, and is characterized by acidic soil and multiple soil types. No major lakes or rivers are located in the area of interest. Environmental conditions at the home sites for patients are shown in Table 2.

**Table 2:** Environmental conditions at human blastomycosis patients' homes, Duplin County, North Carolina, 2001*

Patient	Proximity to nearest major stream, m	Proximity to nearest minor stream, m	Elevation of nearest major stream, m	Elevation of nearest minor stream, m	Soil type
1	4,575.21	498.07	25.48	36.65	Norfolk loamy sand
2	3,047.30	398.11	25.03	32.98	Marvin and Gritney
3	6,602.32	1,073.54	39.36	36.04	Woodington loamy fine sand
4	4,105.22	591.10	44.84	40.82	Rains fine sandy loam
5	3,541.88	767.95	44.84	39.65	Rains fine sandy loam
6	4,773.45	395.70	44.84	39.13	Goldsboro loamy sand

*Home site address was unavailable for 2 case-patients.

The mean county temperatures were 13.8°C for October through December 2001 and 12.1°C for these 3 months from 1996 to 2000. Mean total precipitation was 8.6 cm for October through December 2001 compared with 21.3 cm for these 3 months for 1996 to 2000.

During the fall of 2001, two construction projects were taking place at the school, and crops were harvested in a newly cultivated field near the school. However, nonstudent patients had no known contact with the school grounds. Furthermore, no one with high occupational risk for exposure had symptoms consistent with blastomycosis during the time of interest.

## Conclusions

This investigation underscores the difficulty in identifying the source for a blastomycosis outbreak. Little success has been achieved in isolating *B. dermatitidis* from soil, especially without a potential common exposure site (10). Because the median incubation period for blastomycosis is 30–45 days (11) the environmental conditions at the exposure site often differ between the exposure period and the time of the outbreak investigation. No investigations, including 2 other investigations in North Carolina, have achieved cultural confirmation of an environmental source in the absence of good epidemiologic evidence (7,8). For this reason, environmental testing was not performed in this investigation. Sources suggest that canine blastomycosis might predate human cases (12), but human cases were identified first in this cluster.

Although isolating *B. dermatitidis* from the environment is challenging, certain conditions have been associated with blastomycosis in earlier outbreaks and may have contributed to this one. For example, the acidic pastureland of the area and proximity of patient homes to low-lying waterways are both consistent with sites of other outbreaks (2,4,9). Although excavations have been implicated in previous outbreak investigations of blastomycosis (1,3), the earth-disturbing activities in this outbreak could not account for nonstudent patients. Although most patient home sites were located on soils containing sand, none were pure sand. Humidity and precipitation may encourage release of *B. dermatitidis* spores (1,3). In North Carolina, the average relative humidity does not vary greatly from season to season but is generally highest in winter (13). Precipitation during the months in question was diminished compared with the previous 5 years, and only 3 days of rain were recorded in November 2001.

Diagnostic testing for blastomycosis can be problematic. Because of poor sensitivity and specificity, the skin test antigen blastomycin is not available, and serologic tests also show poor specificity (14). The diagnostic standard is visualization of the yeast form of *B. dermatitidis* in a clinical specimen (11). Culture is ideal, but the organism may take up to 5 weeks to grow (15). Furthermore, obtaining a positive specimen may require invasive techniques. Finally, a user-friendly rapid test to determine population exposure is not available for blastomycosis.

This report illustrates the challenges to investigating blastomycosis clusters. Research suggests that rapid growth of *B. dermatitidis* may be promoted by local environmental conditions, which may have been the case in the present outbreak. However, outbreak investigations of this rare but potentially serious condition would be more conclusive with the ability to isolate the organism from the environment, timely diagnosis of the disease, and availability of a sensitive and specific screening test to assess population exposure.
